# A note on misspecification in general linear models with correlated errors for the analysis of crossover clinical trials

**DOI:** 10.1371/journal.pone.0213436

**Published:** 2019-03-14

**Authors:** Wei Wang, Ning Cong, Tian Chen, Hui Zhang, Bo Zhang

**Affiliations:** 1 Center of Biostatistics and Bioinformatics, University of Mississippi Medical Center, Jackson, MS, United States of America; 2 Department of Surgical Oncology (Interventional Therapy), Shandong Cancer Hospital and Institute, Shandong Academy of Medical Sciences, Jinan, Shandong, P.R. China; 3 Department of Mathematics and Statistics, University of Toledo, Toledo, OH, United States of America; 4 Department of Biostatistics, St. Jude Children’s Research Hospital, Memphis, TN, United States of America; 5 Department of Population and Quantitative Health Sciences, University of Massachusetts Medical School, Worcester, MA, United States of America; The Wistar Institute, UNITED STATES

## Abstract

Among various approaches to the repeated measures analysis in crossover clinical trials, the general linear models (GLMs) with correlated errors attract substantial attention due to their simplicity in model specification, implementation, and interpretation. The goal of this research note is to conduct simulation studies to numerically investigate the impact of model misspecification in the GLMs with correlated errors in the analysis of crossover trials. A series of synthetic two-treatment and three-treatment crossover trials were designed, and simulation studies were conducted to assess how treatment effect estimation, type I error rates, and power can be affected by misspecified period effects, carryover effects, and variance-covariance structures in the GLMs. Numerical studies confirm that (i) the GLMs with terms for both carryover and period effects and with an unstructured variance-covariance matrix can provide unbiased treatment effect estimates and control of Type I error rates and that (ii) misspecification in either period effects, carryover effects, or covariance structures in the GLMs can induce inflated type I error, declined power, or biased treatment effect estimates. Although methodologic contribution of this research note is minimal, we provide practical recommendations and advice to pharmaceutical sponsors and other investigational drugs and device applicants in designing and analyzing crossover trials using the GLMs with correlated errors.

## Introduction

In clinical trials with a crossover design, study subjects are assigned to receive a sequence of different treatments in multiple study periods, during which study endpoints are repeatedly measured in each period on the subjects. The crossover trials are not uncommon in investigations of new medical devices [1] and are standard in drug studies of bioequivalence [2]. Senn [3] and Jones and Kenward [4] provide systematic reviews on design and analysis of crossover trials. Crossover trials have two major advantages over the conventional parallel-group clinical trials. First, the influence of confounding covariates on treatment evaluation can be largely reduced because the crossover subjects essentially serve as their own control. Second, optimal crossover designs are statistically efficient, and therefore fewer subjects are required than the conventional parallel-group designs. However, crossover trials still have substantial issues in data analysis [5]. One of the issues is presence of period effects caused by the order that treatments are given to study subjects. Period effect represents a systematic difference between different periods in the outcome for evaluating a treatment. The presence of a period effect may suggest that a patient’s underlying condition and potential to respond to the treatment would have changed from one treatment period to another. To avoid confounding period effects, groups of subjects are randomized to multiple sequences of treatments [4]. Another issue is potential existence of carryover effects that may affect study endpoints together with the “direct effect” of treatments administered to the subjects. Carryover effect is defined as the lingering effect of the treatment of the previous study period on the current study period. It presents when the treatment effect given in the previous period persists into the second period and distorts the current treatment effect. Carryover effects in crossover trials may bias analysis of the direct treatment effect [6].

Statistical methodologies for analyzing crossover trials were developed for various types of study endpoints, including dichotomous endpoints [7– 8] and ordinal endpoints [9–10], but a large body of literature discusses the methodologies for continuous endpoints [11–12]. For continuous and normally distributed endpoints, Bellavance et al. [13] proposed a modified *F*-test approximation that accounts for the correlations within subjects induced by repeated measures to conduct relevant hypothesis tests. Simulation studies conducted by Bellavance et al. showed that the modified *F*-test approximation gives adequate control of the type I error rate over a variety of the covariance structure for three-period crossover trials [13]. Yet, Jones and Kenward [4] promoted the use of linear fixed-effects and random-effects models to analyze crossover trials. Bellavance and Tardif [14] described a nonparametric approach to analyze the three-treatment, three-period crossover trials by providing unbiased treatment effect estimates and transforming the original crossover design into a randomized block design in which the well-known rank tests can be applied. Öhrvik [15] proposed another nonparametric method that can be applied to a class of crossover trials with three or more treatments.

Among a variety of analysis approaches, general linear models (GLMs) with correlated errors have attracted substantial attention as tools to analyze the data from crossover trials, primarily because of their simplicity in model specification, implementation, and interpretation [4]. However, in practice, model specification of GLMs with correlated errors has caused massive troubles, as data analysts and clinical investigators have had difficulty deciding whether or when they should include period effects and carryover effects in the GLMs and which variance-covariance structure they should assume for the GLMs. For the GLMs, the likelihood-ratio test and information criteria, such as the Akaike information criterion (AIC) and the Bayesian information Criterion (BIC), can be applied to compare the performance of two or more GLMs. Littell at al. [16] showed that specification of covariance structure substantially influences the inference of fixed effects in the analysis of repeated measures data. Lu and Mehrotra [17] recommend using unstructured covariance as the default strategy for analyzing longitudinal data from randomized clinical trials with a moderate-to-large number of subjects and a small-to-moderate number of time points. The goal of this research note is to conduct simulation studies to numerically investigate the impact of model misspecification in the GLMs with correlated errors in the analysis of data collected from crossover clinical trials. This investigation was motivated by two real-world randomized crossover clinical trials for investigational medical devices: a two-treatment, two-period crossover trial comparing a new test contact lens with a control lens and a three-treatment, three-period crossover trial comparing two new contact lenses with a control lens. Consequently, we designed a series of synthetic two-treatment and three-treatment crossover trials and simulated datasets from these trials to assess how treatment effect estimation, type I error, and power for testing treatment effects are affected by misspecified period effects, carryover effects, and variance-covariance structures.

The messages delivered by this research note are concise. The numerical studies confirm that the GLMs including carryover and period effects can provide unbiased treatment effect estimates and control of Type I error rates if carryover effects are identifiable. Additionally, assuming an unstructured covariance structure for the GLMs has proven to be a safe choice if there is not sufficient confidence in covariance structure specification. The numerical studies show that misspecification in either period effects, carryover effects, or covariance structures likely induces inflated type I error, declined power, or biased treatment effect estimates. We recommend adopting the GLMs with carryover and period effects and an unstructured covariance structure to analyze crossover trials. Also, we verified that the balanced crossover design should be preferred over the unbalanced crossover design. Although methodologic contribution of this research note is minimal, its practical contribution is solid and substantial. This note provides recommendations and advice to pharmaceutical sponsors and other investigational drug and device applicants who would use the GLMs with correlated errors as their primary analysis approach to analyze crossover trial data but are confused on model-specification issues. These issues have never been thoroughly addressed in the context of analyzing crossover trials.

## Modeling framework and misspecification

### General linear models with correlated errors for the analysis of crossover clinical trials

We consider an *s*-sequence, *p*-period crossover clinical trial that compares *t* treatments, and it is assumed that in the crossover trial there are *n*_*i*_ subjects in sequence group *i*, *i* = 1,2,⋯,*s*, with $\sum_{i=1}^{s}n_{i}=n$. Let *y*_*ijk*_ denote the response observed on the *k*th subject in period *j* of sequence group *i*, where *j* = 1,2,⋯,*p* and *k* = 1,2,⋯,*n*_*i*_. In this research note, we assume that the response variable *y*_*ijk*_ is continuous and normally distributed and investigate misspecification in the GLM with correlated errors

$$y_{ijk}=\mu +\pi_{j}+\tau_{d[i,j]}+\lambda_{d[i,j-1]}+\epsilon_{ijk}$$ (1)

for analyzing the data collected from such a crossover trial. In (1), *μ* is an intercept, *π*_*j*_ is the effect associated with period *j*, *τ*_*d*[*i*,*j*]_ represents the direct treatment effect associated with the treatment applied in period *j* of sequence *i* with *d*_[*i*,*j*]_ = 1,2,⋯,*t*, *λ*_*d*[*i*,*j*−1]_ denotes the first-order carryover effect from the treatment applied in the preceding period *j*−1 of sequence *i* with *d*_[*i*,*j*−1]_ = 1,2,⋯,*t*, and *λ*_*d*[*i*,0]_ = 0, and *ϵ*_*ijk*_ is the random error with zero mean and variance var(*ϵ*_*ijk*_). In the crossover trial, each subject is repeatedly measured during the *p* periods. Therefore, it is necessary to specify a shared variance-covariance structure $\Sigma =\Sigma_{ik}(cov(\epsilon_{ij_{1}k},\epsilon_{ij_{2}k})$ is its (*j*_1_,*j*_2_) entry) for the GLM (1) to account for the correlated response measurements from each subject. In the analysis of two-period crossover trials, we specify a compound symmetry (CS) covariance structure for Σ. With this specification, (1) is equivalent to a random-intercept model if the covariance components in Σ are non-negative. In the analysis of three-period crossover trials, two covariance structures are considered: compound symmetry and unstructured (UN) covariance structure. Additional terms such as the second-order carryover effect, direct treatment-by-carryover interaction effect, and direct treatment-by-period interaction effect, can be added to (1). However, such terms are rarely of much interest in practice [4] and are not included in our investigation.

The estimation strategy for (1) is to achieve unbiased estimation of both fixed effects and covariance parameters simultaneously by using a likelihood function. For a specified covariance structure Σ, the maximum likelihood (ML) estimator for the fixed effects is the generalized least squares (GLS) estimator. If we assume the response variable *y*_*ijk*_ in (1) normally distributed, let $Y={\left(y_{111},\ldots ,y_{1p1},\ldots ,y_{spn_{s}}\right)}^{\prime }$ and let *X* represent the design matrix, the GLS estimator of fixed effects vector *β* is

$$\hat{\beta }={(X\prime (\Sigma }^{-1}⨂I_{n})X{)}^{-1}X\prime (\Sigma^{-1}⨂I_{n})Y$$ (2)

where ⨂ represents the Kronecker product. Asymptotically,

$$\hat{\beta }∼N(\beta ,(X\prime (\Sigma^{-1}⨂I_{n})X{)}^{-1})$$

If Σ is known, the GLS estimator (2) would be a best linear unbiased estimator (BLUE). However, Σ is usually unknown in practice. We then estimate the parameter by substituting the unknown Σ with its estimate $\hat{\Sigma }$,

$${\hat{\beta }}_{E}=(X\prime ({\hat{\Sigma }}^{-1}⨂I_{n})X{)}^{-1}X\prime ({\hat{\Sigma }}^{-1}⨂I_{n})Y$$ (3)

For a specified structure pattern for Σ, ${\hat{\beta }}_{E}$ in (3) can be estimated using the maximum likelihood (ML) method with a reduced log-likelihood [18]. However, Diggle et al. [18] noted that the ML estimation presents with conflict because a large design matrix is needed for consistent estimates, whereas a design matrix with a small number of columns is required to yield approximately unbiased estimation. The method of restricted maximum likelihood (REML) [19] is usually applied as the objection to the ML procedure that can produce biased estimators for covariance parameters [20]. Swallow and Monahan [21] recommend the REML method over other variance component estimation methods on the basis of the results from their simulation studies. The REML method for covariance parameters estimation is used throughout this article, combined with an adjustment procedure developed by Kenward and Roger [22]. This procedure had been proved with a notable effect in controlling type I errors in small sample size studies [22]. Inference based on this combined procedure is more reliable than others for analyzing crossover trials [11].

### Misspecification of carryover and period effects

Model misspecification remains a critical issue for the data analysis of crossover clinical trials because misspecified models can create bias in treatment effect estimation and the corresponding hypothesis testing. Here, we investigate the impact of misspecification in the GLMs with correlated errors in the analysis of data collected from crossover clinical trials. The primary objective of our investigation is to gauge the extent of inference bias on treatment effects when carryover effect, period effect, or both are omitted in such models. Considered in this research note are four analysis approaches, including three GLMs with distinct mean structures and a naïve hypothesis testing procedure: the GLM (1) is the approach that is considered with both period effect and carryover effect, and it is abbreviated as the “PE-CE model”. The GLM that only includes period effect is specified as

$$y_{ijk}=\mu +\pi_{j}+\tau_{d[i,j]}+\varepsilon_{ijk}$$ (4)

and is abbreviated as the “PE-NCE model”; the GLM that does not include either period effect or carryover effect is specified as

$$y_{ijk}=\mu +\tau_{d[i,j]}+\varepsilon_{ijk}$$ (5)

and is abbreviated as the “NPE-NCE model”. The naïve hypothesis testing procedure refers to the approach of treating only the measurements from the first period as a randomized, parallel study and ignoring other periods (i.e., periods 2 to *p*). For a two-period crossover trial assessing two treatment groups, the two-sample *t*-test is used; for a three-period crossover design, the one-way analysis of variance, or ANOVA, is used to determine the treatment effects.

### Motivation: Two crossover clinical trials for investigational medical devices

Contact lenses are medical devices used to provide flexible and convenient vision correction. Contact lenses can be used to correct various vision disorders, including myopia, hyperopia, presbyopia, and astigmatism. The numerical investigation reported in this research note was motivated by two real-world randomized crossover clinical trials for investigational contact lenses: a two-treatment, two-period crossover trial comparing a new test contact lens with a control lens and a three-treatment, three-period crossover trial comparing two new contact lenses with a control lens. Both clinical trials are balanced design, enrolling 48 subjects for the two-period, two-sequence trial (24 subjects in each of the 2 sequences: CT and TC; “C” represents the control lens and “T” represents the test lens) and 18 subjects for the three-period trial (3 subjects in each of the 6 sequences: CT_1_T_2_, CT_2_T_1_, T_1_CT_2_, T_1_T_2_C, T_2_CT_1_ and T_2_T_1_C; “C” represents the control lens, “T_1_” represents the first test lens, and “T_2_” represents the second test lens). The primary endpoint of both trials was the subjective visual quality scores for the test versus control lenses on a scale from 0 to 100, with 0 denoting unfavorable visual quality. Boundary issues on the primary endpoint were ignored in the analysis.

For the two crossover trials, Table 1 displays and compares the estimated treatment effects, period effects, and first-order carryover effects and their standard errors obtained from the PE-CE model, the PE-NCE model, the NPE-NCE model, with CS and UN covariance structures, and from the naïve hypothesis testing procedure (two-sample *t*-test or one-way ANOVA) that only tests the outcome measurements from the first study period. Throughout this note, the treatment effect refers to the direct effect of a treatment minus the direct effect of its control, and thus, is distinguished with the direct treatment effects defined in (1).

**Table 1:** Comparison of estimated treatment effects, period effects, and first-order carryover effects and their standard errors (in parentheses) given by the three GLMs (with CS and UN covariance structures) and naïve hypothesis testing procedure for real-world two-period and three-period crossover trials.

	Test Lens 1		Test Lens 2		Period Effects	
	Treatment Effect (SE)	Carryover Effect (SE)	Treatment Effect (SE)	Carryover Effect (SE)	Period 2 Effect (SE)	Period 3 Effect (SE)
**Two-Period Crossover Trial**						
Two-sample *t*-test	24.6 (7.4)	-	-	-	-	-
PE-CE model	24.6 (7.7)	9.5 (12.9)	-	-	-3.8 (7.7)	-
PE-NCE model	19.9 (4.2)	-	-	-	1.0 (4.2)	-
NPE-NCE model	19.9 (4.2)	-	-	-	-	-
**Three-Period Crossover Trial**						
One-way ANOVA	6.2 (6.3)	-	6.7 (6.3)	-	-	-
*Compound Symmetry* Σ						
PE-CE model	1.0 (3.1)	-1.3 (4.0)	1.5 (3.1)	-0.8 (4.0)	0.1 (3.6)	0.6 (3.6)
PE-NCE model	1.4 (2.7)	-	1.7 (2.7)	-	-0.6 (2.7)	-0.1 (2.7)
NPE-NCE model	1.4 (2.6)	-	1.7 (2.6)	-	-	-
*Unstructured* Σ						
PE-CE model	0.7 (3.1)	0.3 (4.4)	2.6 (3.1)	-0.9 (4.4)	-0.3 (3.8)	0.1 (3.4)
PE-NCE model	0.7 (2.9)	-	2.9 (2.9)	-	-0.6 (2.8)	-0.1 (2.3)
NPE-NCE model	0.6 (2.8)	-	2.8 (2.8)	-	-	-

For the two-period crossover trial, four analysis approaches all suggested significantly higher visual quality scores for the test lens than for the control lens. The estimates of the treatment effect and corresponding standard errors obtained from the naïve two-sample *t*-test and the PE-CE model were almost identical. Substantial carryover effect (approximately 40% of treatment effect) and a modest period effect (approximately -15% of main treatment effect) were detected from the PE-CE model, although neither effect was statistically significant. The treatment effect estimates obtained from the PE-NCE model and the NPE-NCE model were similar, and these treatment effect estimates were distinct from those obtained from the naïve two-sample *t*-test and the PE-CE model.

For the three-period crossover trial, the visual quality scores of two new test lenses and those of the control lens were similar in all four analysis approaches. The effect estimates differed when specifying a CS or UN covariance structure for the GLMs. The treatment effect estimates given by the one-way ANOVA procedure and the PE-CE model were not comparable. This dissimilarity may be due to the small sample size, large between-subject variation, or the higher-order carryover effects that were not considered in the analysis. The treatment effect estimates and corresponding standard errors given by the three GLMs were close to but still distinct from each other.

Analysis results in Table 1 reveal that the GLMs with different model specification can result in uncertainty in treatment effect estimation and hypothesis-testing conclusions. Therefore, model misspecification remains an issue that will largely affect the use of the GLMs with correlated errors in the analysis of crossover trials. This fact motivated us to investigate the impact of model misspecification in the GLMs in such an analysis task. A series of simulation studies were designed and conducted to assess whether treatment effect estimation, as well as type I error and power in hypothesis testing, will be affected by misspecified period effects, carryover effects, and variance-covariance structures in the GLMs with correlated errors.

## Simulation studies

### Two-period, two-treatment crossover trials

We designed and conducted two simulation studies to investigate the impact of misspecification of period and carryover effects in the GLMs with correlated errors for analyzing the data collected from two-period, two-treatment (two-by-two) crossover trials. It was assumed that, in the two-by-two crossover trials, a treatment (denoted by “T”) and a control (denoted by “C”) were compared through two sequences, TC and CT.

### Type I error under true null hypotheses

To investigate the impact of misspecification of period and carryover effects on Type I error obtained from testing treatment effects, 2000 datasets were generated from the GLM (1), with sample sizes of *n* = 20 and *n* = 200 and with both a balanced design (CT:TC = 1:1) and an unbalanced design (CT:TC = 1:3). When generating the datasets, standard deviation of the response variable was fixed, and then three sets of period effects (period 2 relative effect) were considered as −15%, 0%, or 25% of the response standard deviation. Under the true null hypotheses that no treatment or carryover effects exist (when the treatment effect is zero, it was reasonable to assume the corresponding carryover effect is zero as well), both the treatment effects and carryover effect differences were set to be zero. Then, the response values were generated according to the GLM (1) with a CS covariance matrix using the within-subject correlation coefficient 0.2, 0.5, or 0.7, respectively.

**Fig 1:**
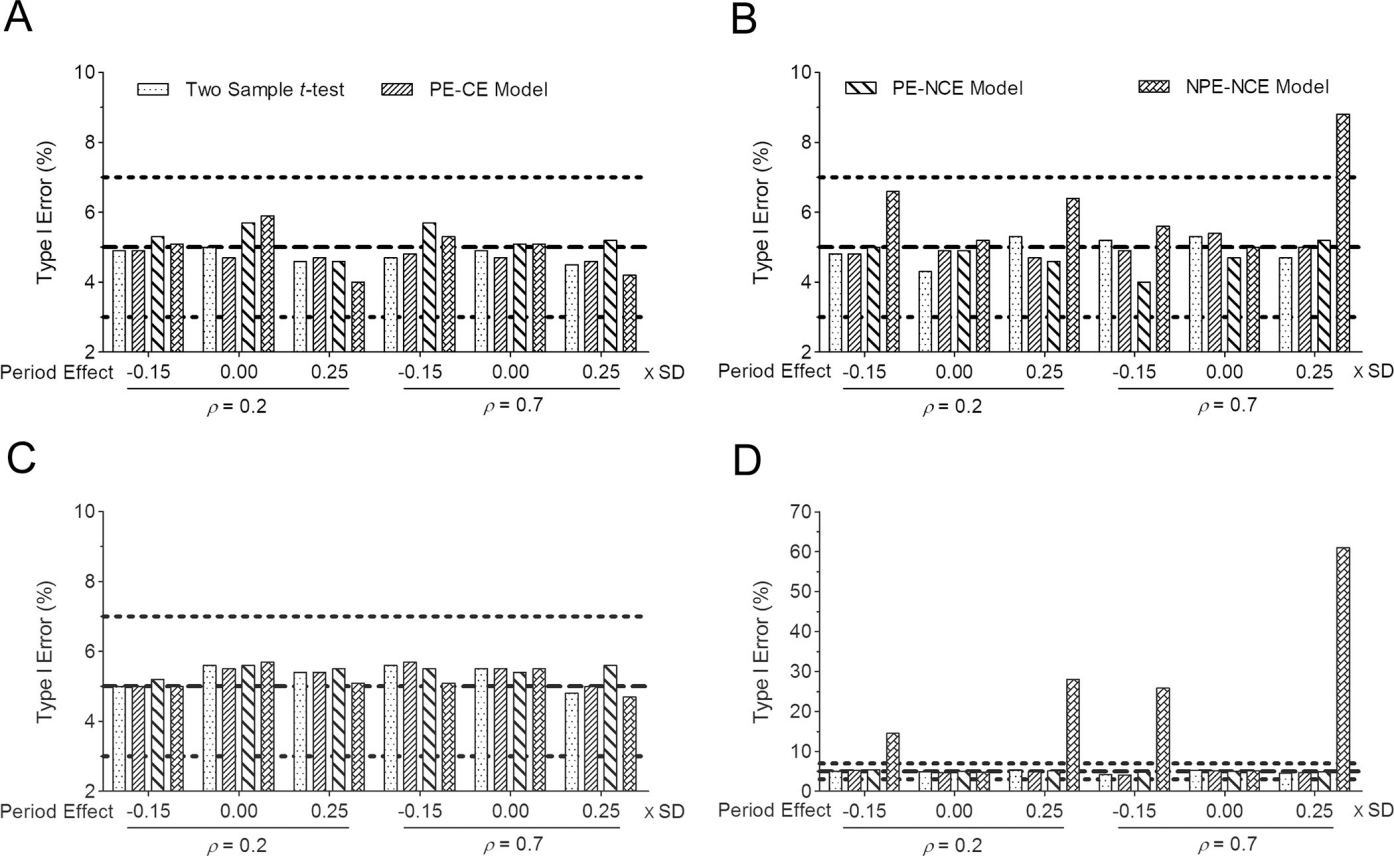
Type I error rates for the analysis of two-period, two-treatment crossover trials based on 2000 simulated data sets for the balanced (A, *n* = 20; C, *n* = 200) and unbalanced (B, *n* = 20; D, *n* = 200) designs.

For each dataset, the four analysis approaches (two-sample *t*-test that only analyzes the outcome measurements from the first study period, the PE-CE model, the PE-NCE model, and the NPE-NCE model) were used to estimate the treatment effect and test whether the treatment effect was equal to zero. The Wald test was conducted in the hypothesis testing of treatment effects with the GLMs. The empirical type I error rates of the four approaches obtained in different scenarios are summarized in Fig 1. All four approaches yielded type I error rates near the nominal level of 5% (Fig 1A and 1C) when analyzing the data simulated from the balanced crossover trials, regardless of sample sizes (small or large) and period effects (zero or not). For the data simulated from the unbalanced crossover trials, the type I error rates obtained from the two-sample *t*-test, the PE-CE model, and the PE-NCE model were still within 5% of the nominal level. However, the NPE-NCE model produced noticeably inflated type I error rates when the period effect existed (Fig 1B and 1D), especially with a large sample size.

### Power and estimation bias

To investigate the impact of misspecification of period effects, carryover effects, and covariance structures on estimation of treatment effect and power obtained in testing treatment effects, 2000 datasets were generated from the two-by-two crossover trials as described above but with nonzero treatment and carryover effects. In this simulation study, the treatment effect was fixed at the size of 50% of the response standard deviation, and the carryover effect difference was 0%, 15%, or 25% of the response standard deviation. This is equivalent to assuming that 0%, 30%, or 50% of the treatment effect was carried over from the first to the second period.

For each generated dataset, the four analysis approaches were used to estimate the treatment effect and to construct a 95% confidence interval for the treatment effect. We calculated the average percent error (PE = 100 × (Estimated Treatment Effect−True Treatment Effect)/True Treatment Effect) that quantifies the estimation bias of the treatment effect, the power (the percentage of simulated datasets for which the 95% confidence intervals of the treatment effect estimates did not cover zero), and the coverage probability (CP, the percentage of simulated datasets for which the 95% confidence intervals of the treatment effect estimate covered the true value). Table 2 and Table 3 summarize the estimation bias quantified by the PE, the power, and the CPs of the 95% confidence intervals obtained by analyzing the datasets for the balanced and unbalanced two-by-two crossover trials with the small sample size *n* = 20. Simulation results obtained from the large sample size *n* = 200 were similar, and thus are not reported here.

**Table 2:** Average percent error, power, and 95% coverage probabilities obtained from the analysis of two-period, two-treatment crossover trials based on 2000 simulated datasets, balanced design, sequence CT:TC = 1:1, sample size *n* = 20, and effect size 0.5.

*ρ*	Period Effect (%)^†^	Carryover Effect (%)^†^	Two-sample t-test			PE-CE model			PE-NCE model			NPE-NCE model		
			PE (%)	Power (%)	CP (%)	PE (%)	Power (%)	CP (%)	PE (%)	Power (%)	CP (%)	PE (%)	Power (%)	CP (%)
0.2	-15	0	1.2	17.8	95.2	1.2	18.9	94.9	-1.6	37.4	94.8	-1.6	37.4	95.3
	-15	15	0.8	18.9	94.8	0.8	19.9	94.7	-15.0	31.5	94.1	-15.0	31.5	94.1
	-15	25	1.2	18.9	95.1	1.2	19.9	95.2	-24.8	25.8	91.2	-24.8	25.6	91.4
	0	0	-2.1	18.7	95.6	-2.1	18.9	95.3	-1.3	40.4	95.2	-1.3	39.8	95.3
	0	15	2.0	19.1	95.4	2.0	20.1	95.2	-13.7	31.2	94.7	-13.7	31.3	94.6
	0	25	-2.4	17.2	95.4	-2.4	17.8	95.3	-24.2	25.4	93.7	-24.2	25.2	93.6
	25	0	-0.3	18.2	95.7	-0.3	19.4	95.2	0.2	40.4	95.1	0.2	38.5	95.7
	25	15	0.8	20.1	95.2	0.8	19.8	95.0	-15.5	30.2	93.6	-15.5	28.3	94.0
	25	25	1.0	18.6	95.3	1.0	19.3	95.4	-24.3	25.2	93.2	-24.3	23.1	94.3
0.5	-15	0	-2.2	18.9	95.0	-2.2	19.6	94.7	-1.4	54.6	94.6	-1.4	53.4	94.7
	-15	15	-3.9	18.1	95.1	-3.9	18.4	95.0	-17.5	40.9	92.9	-17.5	40.9	93.4
	-15	25	1.5	19.9	95.1	1.5	20.1	95.5	-24.0	37.4	91.9	-24.0	37.8	91.7
	0	0	2.4	18.6	94.9	2.4	19.8	95.3	0.5	55.9	94.7	0.5	56.4	94.9
	0	15	-1.4	18.3	95.3	-1.4	18.8	94.9	-15.6	42.8	94.3	-15.6	43.0	94.6
	0	25	1.6	19.0	94.9	1.6	19.6	94.6	-25.4	36.0	91.6	-25.4	35.3	91.7
	25	0	2.4	20.0	95.3	2.4	19.9	95.5	-0.5	56.8	95.4	-0.5	54.9	95.9
	25	15	-0.6	19.1	95.1	-0.6	20.2	94.9	-14.8	43.5	93.3	-14.8	39.9	94.8
	25	25	-1.8	18.1	95.1	-1.8	18.6	94.9	-25.3	36.5	91.2	-25.3	31.7	93.4
0.7	-15	0	-1.5	17.6	96.1	-1.5	18.1	96.0	0.2	76.9	95.0	0.2	76.0	95.2
	-15	15	1.0	17.5	95.5	1.0	19.0	95.3	-14.9	63.9	92.4	-14.9	64.1	92.7
	-15	25	-1.9	18.3	95.3	-1.9	18.5	95.4	-24.7	54.5	89.6	-24.7	54.7	89.5
	0	0	0.5	19.4	95.1	0.5	20.4	95.0	-0.0	78.9	95.1	-0.0	78.8	95.2
	0	15	0.4	17.7	95.9	0.4	18.4	95.6	-13.7	65.7	93.8	-13.7	65.4	94.0
	0	25	-3.6	17.3	94.5	-3.6	17.8	94.6	-24.1	54.6	89.7	-24.1	54.3	90.1
	25	0	1.1	19.9	94.9	1.1	20.4	94.6	1.4	78.3	94.9	1.4	75.6	95.4
	25	15	-0.4	18.6	95.2	-0.4	19.3	95.5	-16.5	62.8	93.3	-16.5	54.9	94.9
	25	25	-2.6	17.8	95.1	-2.6	18.6	95.1	-26.1	52.7	89.0	-26.1	44.0	92.3

^†^ Proportion of response standard deviation.

**Table 3:** Average percent error, power, and 95% coverage probabilities obtained from the analysis of two-period, two-treatment crossover trials based on 2000 simulated datasets, unbalanced design, sequence CT:TC = 1:3, sample size *n* = 20, and effect size 0.5.

ρ	Period Effect (%)^†^	Carryover Effect (%)^†^	Two-sample t-test			PE-CE model			PE-NCE model			NPE-NCE model		
			PE (%)	Power (%)	CP (%)	PE (%)	Power (%)	CP (%)	PE (%)	Power (%)	CP (%)	PE (%)	Power (%)	CP (%)
0.2	-15	0	0.7	14.0	94.6	0.7	15.2	95.3	0.1	31.9	94.6	15.0	50.1	93.3
	-15	15	-4.2	14.9	94.5	-4.2	14.5	94.6	-17.3	24.0	93.6	-9.2	34.0	94.5
	-15	25	-1.5	14.8	94.3	-1.5	14.7	95.3	-25.5	19.1	92.9	-22.8	24.3	93.0
	0	0	2.1	15.7	95.1	2.1	15.6	94.8	1.1	33.4	94.5	0.0	41.4	94.9
	0	15	4.5	16.5	95.5	4.5	17.1	96.0	-14.3	24.5	94.1	-23.2	26.0	93.5
	0	25	0.8	14.6	94.9	0.8	14.9	95.1	-25.8	20.8	93.3	-38.3	18.1	90.3
	25	0	-2.8	15.0	95.5	-2.8	14.9	95.7	-0.1	32.7	94.6	-24.7	24.8	92.2
	25	15	-4.8	14.6	94.1	-4.8	14.7	94.4	-16.0	23.9	94.4	-47.7	13.5	88.2
	25	25	-1.5	15.3	94.5	-1.5	15.6	94.9	-25.5	19.0	92.6	-62.5	9.4	82.6
0.5	-15	0	3.6	16.6	94.5	3.6	17.7	94.8	2.1	46.5	95.0	16.8	71.0	93.7
	-15	15	0.5	15.5	95.6	0.5	15.5	96.3	-14.0	35.9	94.0	-7.3	50.5	95.1
	-15	25	0.1	15.4	94.6	0.1	15.8	94.4	-22.6	29.1	92.9	-21.3	39.6	91.5
	0	0	-1.6	14.8	94.8	-1.6	14.4	95.6	0.1	46.4	94.6	-0.4	56.2	95.7
	0	15	-2.4	14.7	94.0	-2.4	14.4	94.2	-13.9	35.5	93.8	-21.2	38.0	92.9
	0	25	-2.8	15.0	94.3	-2.8	15.2	94.0	-26.4	26.5	93.2	-39.3	25.1	86.8
	25	0	2.1	16.2	93.9	2.1	15.8	94.3	1.1	46.0	95.3	-23.8	34.9	92.6
	25	15	5.1	17.2	95.0	5.1	17.3	95.0	-15.5	35.2	94.5	-48.4	17.9	84.2
	25	25	4.0	17.3	95.3	4.0	17.0	95.3	-24.0	28.8	93.1	-61.5	12.5	76.0
0.7	-15	0	0.3	14.8	95.4	0.3	15.2	95.6	-1.2	65.1	95.1	14.4	86.6	94.7
	-15	15	-4.7	14.5	94.1	-4.7	14.5	94.4	-15.5	51.0	94.1	-7.7	71.5	95.4
	-15	25	-3.3	13.8	95.4	-3.3	14.5	95.4	-25.8	41.6	91.1	-22.8	56.5	90.9
	0	0	-6.9	14.4	95.6	-6.9	13.7	96.1	-0.9	66.4	95.4	-0.5	78.3	94.7
	0	15	3.3	15.5	94.1	3.3	15.5	94.1	-14.3	52.4	94.0	-21.8	56.6	91.2
	0	25	4.0	15.2	95.5	4.0	16.1	95.6	-23.8	43.8	91.3	-37.0	39.2	83.3
	25	0	4.1	15.7	95.2	4.1	14.6	95.5	0.3	65.5	96.0	-25.2	49.7	91.6
	25	15	-1.3	14.7	94.4	-1.3	14.6	94.6	-15.1	52.1	92.5	-47.8	26.0	76.7
	25	25	-2.4	14.9	95.1	-2.4	14.9	95.2	-25.9	42.5	90.9	-63.1	13.0	64.9

^†^ Proportion of response standard deviation.

For the two-by-two crossover trials with a balanced design (Table 2), the two-sample *t*-test and the PE-CE model produced similar unbiased estimators. The PE-NCE model and the NPE-NCE model gave treatment effect estimates that were close to each other but were biased when carryover effects existed, and the bias was proportional to the magnitude of the carryover effects. The two-sample *t*-test generated power similar to that of the PE-CE model, and the power was stable as the period effect, carryover effect, and within-subject correlation coefficient changed. The PE-NCE model and the NPE-NCE model had larger power than did the two-sample *t*-test or the PE-CE model. The power remained stable with different magnitudes of the period effect, but decreased as carryover effect increased, and increased as the within-subject correlation coefficient increased. For the two-by-two crossover trials with an unbalanced design (Table 3), the two-sample *t*-test and the PE-CE model still produced comparable unbiased estimators and power. However, estimation bias of the treatment effect and the power obtained from using the PE-NCE model and the NPE-NCE model were not close anymore. Treatment effect estimates remained biased when carryover effects existed for the PE-NCE model and the NPE-NCE model when analyzing the unbalanced two-by-two crossover trials. The NPE-NCE model produced larger relative bias than did the PE-NCE model with up to 60% PE, when substantial period effect and carryover effect existed.

### Three-period, three-treatment crossover trials

We designed and conducted two more simulation studies to investigate the impact of misspecification of period and carryover effects, as well as covariance structures, in the GLMs with correlated errors for analyzing the data collected from three-period, three-treatment crossover trials. It was assumed that, in the three-period, three-treatment crossover trials, two treatments (denoted by “T_1_” and “T_2_”, respectively) and a control (denoted by “C”) were compared through six sequences: CT_1_T_2_, CT_2_T_1_, T_1_CT_2_, T_1_T_2_C, T_2_CT_1_, and T_2_T_1_C.

### Type I error under the true null hypotheses

To investigate the impact of misspecification of period and carryover effects and covariance structures on Type I error obtained from testing treatment effects, 2000 datasets were generated from the GLM (1) with sample sizes of *n* = 24 for balanced three-period, three treatment crossover trials (*n*/6 subjects in each of the six sequences) and *n* = 240 for unbalanced three-treatment crossover trials (CT_1_T_2_:CT_2_T_1_:T_1_CT_2_:T_1_T_2_C: T_2_CT_1_:T_2_T_1_C = 4:4:1:1:1:1). When generating the datasets, standard deviation of the response variable was fixed, and then three sets of (Period 2 effect, Period 3 effect) combinations were considered as (−6%,−15%), (0%,0%), or (10%,25%) of Period 3 standard deviation. Given the assumed true null hypotheses on treatment effects, it was assumed that both the treatment effects and carryover effect differences were zero. Then, the response values were generated according to the GLM (1) with two CS covariance matrices using the within-subject correlation coefficient of 0.2 or 0.7, a Toeplitz (TP) covariance matrix representing homogeneous variance and distinct pairwise correlation coefficients at three periods, and an unstructured (UN) covariance matrix (specification of the covariance matrices are illustrated in Table 4).

**Table 4:** Variance-covariance matrices specified in generating simulation datasets for the three-period, three-treatment crossover trials.

Compound Symmetry (1)			Compound Symmetry (2)			Toeplitz			Unstructured		
625	125	125	625	437.5	437.5	625	500	208.1	125	125	69.9
125	625	125	437.5	625	437.5	500	625	339.4	125	500	279.5
125	125	625	437.5	437.5	625	208.1	625	625	69.9	279.5	625

**Fig 2:**
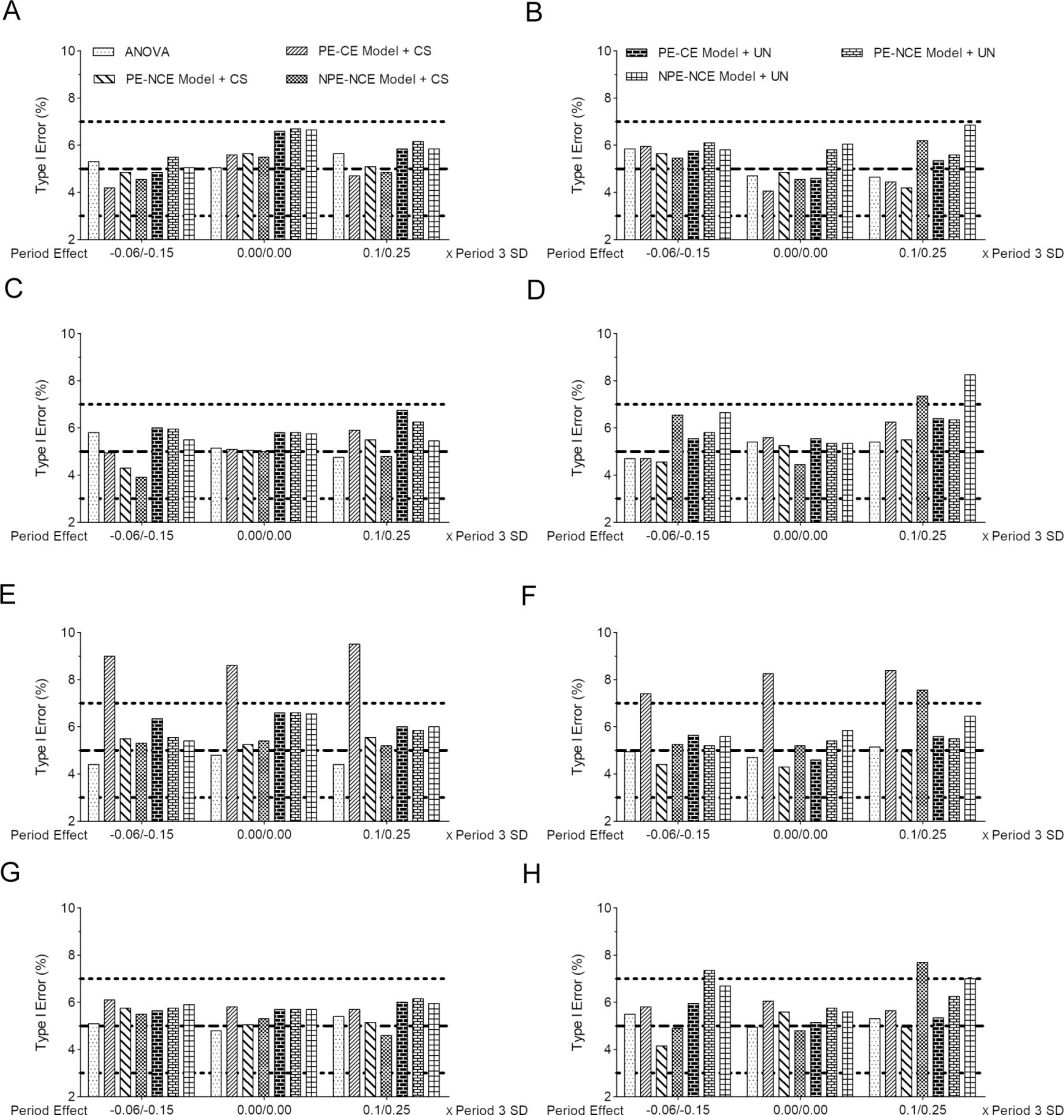
Type I error rates for the analysis of three-period, three-treatment crossover trials based on 2000 simulated data sets for the balanced (A, compound symmetry covariance with *ρ* = 0.2; C, compound symmetry covariance with *ρ* = 0.7; E, Toeplitz covariance; G, unstructured covariance) and unbalanced (B, compound symmetry covariance with *ρ* = 0.2; D, compound symmetry covariance with *ρ* = 0.7; F, Toeplitz covariance; H, unstructured covariance) designs with *n* = 24.

For each dataset, one-way ANOVA that only analyzes the outcome measurements from the first study period and three GLMs (the PE-CE model, the PE-NCE model, and the NPE-NCE model) with both CS and UN covariance structures were used to estimate the treatment effects and to test whether the treatment effect of T_1_ was zero, given that the treatment effect of T_2_ was negligible. Additional simulation studies showed that the magnitude of treatment effect of T_2_ had little impact on estimates, type I error rates, and power of the treatment effect of T_1_ (results are not shown). Fig 2 shows the empirical type I error rates of the four analysis approaches for testing the treatment effect of T_1_ with the small sample size *n* = 24. When the sample size increased to 240, the patterns of type I error rates were unchanged, and therefore, are not shown here. For the balanced three-treatment crossover trials, both one-way ANOVA and three GLMs with the UN within-subject covariance structure maintained an adequate control of the type I error level (Fig 2A, 2C, 2E, and 2G). The three general models with the CS covariance structure performed well in terms of type I error for the datasets that were simulated from the true CS and UN covariance structures (Fig 2A, 2C and 2G). However, the PE-CE model tended to have an inflated type I error rate for the datasets simulated from the true TP covariance structure (Fig 2E). The inflation did not improve as the sample size increased to 240 (results are not shown). For the unbalanced three-treatment crossover trials, the ANOVA method and the PE-CE model with the UN covariance structure yielded type I error rates near the nominal 5% of the datasets that were simulated from different true covariance structures (Fig 2B, 2D, 2F, and 2H). In contrast, three GLMs with the CS covariance structure did not maintain control of type I error rates when the covariance structure was misspecified (Fig 2F).

### Power and estimation bias

To investigate the impact of misspecification of period effects, carryover effects, and covariance structures on estimation of treatment effects and power obtained in testing treatment effects, 2000 datasets were generated from the three-treatment crossover trials as described above, but with nonzero treatment and carryover effects of T_1_. The T_1_ treatment effect was fixed at the size of 50% of the Period 3 standard deviation, and its carryover effect difference was set up as 0% or 25% of the Period 3 standard deviation, which is equivalent to assuming that 0% or 50% of the T_1_ treatment effect was carried over from one period to another. The treatment effect of T_2_ and its carryover effect difference were assumed to be zero. For each dataset, one-way ANOVA and three GLMs (the PE-CE model, the PE-NCE model, and the NPE-NCE model) with both CS and UN covariance structures were used to estimate the treatment effect of T_1_ and to construct a 95% confidence interval for the treatment effect. Table 5 and Table 6 present the estimation bias quantified by the PE, the power, and the CPs of the 95% confidence intervals from the balanced and unbalanced three-treatment crossover trials with small sample size. Simulation results obtained from the large sample size *n* = 240 were similar and are not reported here.

**Table 5:** Average percent error, power, and 95% coverage probabilities obtained from the analysis of three-period, three-treatment crossover trials based on 2000 simulated datasets, balanced design, sequence CT_1_T_2_:CT_2_T_1_:T_1_CT_2_:T_1_T_2_C: T_2_CT_1_:T_2_T_1_C = 1:1:1:1:1:1, sample size *n* = 24, and an effect size of 0.5 for T_1_ and 0 for T_2_.

Period Effect (%)^†^	Carryover Effect (%)^‡^	One-Way ANOVA		GLMs with CS						GLMs with UN					
				PE-CE model		PE-NCE model		NPE-NCE model		PE-CE model		PE-NCE model		NPE-NCE model	
		PE (%)	Power (%)	PE (%)	Power (%)	PE (%)	Power (%)	PE (%)	Power (%)	PE (%)	Power (%)	PE (%)	Power (%)	PE (%)	Power (%)
Compound Symmetry *ρ* = 0.2															
-6/-15	0/0	0.2	16.2	0.9	41.8	1.3	49.3	1.3	49.3	1.1	37.8	1.6	44.7	1.6	44.9
-6/-15	25/0	0.9	16.3	0.6	41.7	-15.0	36.6	-15.0	36.7	0.5	38.4	-15.3	34.2	-15.3	33.8
0/0	0/0	-1.4	15.7	0.2	40.1	-0.2	46.9	-0.2	46.9	-0.6	36.8	-1.2	42.2	-1.4	42.0
0/0	25/0	-2.3	15.7	-0.8	40.1	-17.1	34.0	-17.1	33.7	-2.1	35.4	-18.7	30.0	-18.7	29.7
10/25	0/0	-1.0	15.6	-0.4	40.8	-1.1	47.3	-1.1	46.5	-0.1	37.2	-0.9	43.6	-0.8	43.7
10/25	25/0	1.1	16.6	0.0	40.3	-16.2	34.4	-16.2	33.0	-0.1	36.5	-16.2	32.3	-16.8	30.5
Compound Symmetry *ρ* = 0.7															
-6/-15	0/0	-0.1	17.1	-0.6	77.7	-0.5	85.4	-0.5	85.1	-0.2	73.7	-0.4	81.0	-0.3	81.3
-6/-15	25/0	0.1	17.1	0.9	80.6	-16.4	71.5	-16.4	71.0	1.2	75.9	-16.2	67.2	-16.2	67.1
0/0	0/0	-1.8	14.8	-0.1	79.2	-0.2	87.9	-0.2	88.0	-0.1	73.8	0.0	83.5	-0.1	83.5
0/0	25/0	-2.7	15.2	0.9	79.4	-16.3	71.9	-16.3	71.8	0.9	74.7	-16.3	66.8	-16.5	67.0
10/25	0/0	-0.4	15.7	-1.4	78.6	-0.9	87.0	-0.9	85.9	-1.8	72.6	-1.2	82.8	-1.1	81.8
10/25	25/0	3.5	16.0	3.3	80.8	-14.3	74.3	-14.3	71.3	3.2	77.1	-14.4	68.7	-16.0	64.5
Toeplitz Covariance															
-6/-15	0/0	-3.2	15.8	0.4	62.4	-0.1	71.5	-0.1	71.0	1.1	59.9	-0.4	86.0	-0.3	86.4
-6/-15	25/0	1.2	16.0	-1.9	61.4	-18.4	54.9	-18.4	54.6	-1.7	59.6	-26.9	61.2	-26.9	61.5
0/0	0/0	1.5	17.1	1.0	62.5	0.9	73.1	0.9	73.1	1.4	61.9	0.9	87.2	0.9	87.1
0/0	25/0	-0.7	16.0	0.4	62.2	-16.7	56.5	-16.7	56.3	2.6	61.6	-24.3	64.9	-24.3	64.6
10/25	0/0	5.3	17.4	1.2	62.6	0.7	72.8	0.7	71.9	0.7	59.9	0.0	86.0	-0.1	85.8
10/25	25/0	2.8	16.5	0.8	62.5	-16.2	57.0	-16.2	55.2	1.4	60.8	-25.5	62.5	-26.0	60.3
Unstructured Covariance															
-6/-15	0/0	-1.1	54.8	2.6	68.7	2.0	76.4	2.0	76.2	0.9	80.6	0.9	90.6	0.9	90.6
-6/-15	25/0	0.9	56.7	-0.4	65.9	-17.3	58.6	-17.3	58.5	0.0	80.5	-15.3	77.5	-15.3	77.6
0/0	0/0	1.7	56.8	-1.0	65.5	-0.3	75.5	-0.3	75.7	0.5	80.1	1.2	90.9	1.2	91.2
0/0	25/0	1.6	57.2	0.8	66.5	-15.8	60.1	-15.8	59.9	0.8	79.6	-14.2	79.2	-14.2	78.9
10/25	0/0	0.0	55.7	-2.0	64.2	-1.6	72.6	-1.6	71.4	-0.9	77.8	-0.6	89.2	-0.6	88.9
10/25	25/0	-0.9	55.4	0.1	66.2	-16.5	59.5	-16.5	57.1	-0.2	79.7	-14.9	80.2	-15.3	78.6

^†^ Period 2/Period 3 effects as proportions of Period 3 standard deviation.

^‡^ T_1_/T_2_ carryover effects as proportions of Period 3 standard deviation.

**Table 6:** Average percent error, power, and 95% coverage probabilities obtained from the analysis of three-period, three-treatment crossover trials based on 2000 simulated datasets, unbalanced design, sequence CT_1_T_2_:CT_2_T_1_:T_1_CT_2_:T_1_T_2_C: T_2_CT_1_:T_2_T_1_C = 4:4:1:1:1:1, sample size *n* = 24, and an effect size of 0.5 for T_1_ and 0 for T_2_.

Period Effect (%)^†^	Carryover Effect (%)^‡^	One-Way ANOVA		GLMs with CS						GLMs with UN					
				PE-CE model		PE-NCE model		NPE-NCE model		PE-CE model		PE-NCE model		NPE-NCE model	
		PE (%)	Power (%)	PE (%)	Power (%)	PE (%)	Power (%)	PE (%)	Power (%)	PE (%)	Power (%)	PE (%)	Power (%)	PE (%)	Power (%)
Compound Symmetry *ρ* = 0.2															
-6/-15	0/0	1.3	11.9	-0.8	32.2	-1.1	38.5	-11.1	40.4	-0.5	31.2	-0.5	35.3	-10.7	37.3
-6/-15	25/0	3.4	13.9	0.8	35.2	-17.2	30.2	-18.3	34.0	0.8	33.5	-17.1	27.9	-18.2	32.4
0/0	0/0	-4.1	13.3	0.5	35.2	0.6	40.0	0.4	48.7	0.5	32.2	0.2	36.3	0.1	44.3
0/0	25/0	2.8	13.9	0.9	34.8	-16.5	29.9	-7.7	42.4	1.5	32.6	-16.2	28.8	-7.6	38.7
10/25	0/0	3.6	14.7	0.6	34.5	0.4	39.3	17.4	59.1	1.1	31.5	0.4	36.2	17.2	56.2
10/25	25/0	3.3	13.7	0.0	34.3	-17.8	28.6	8.8	52.9	-0.2	31.8	-18.2	26.9	7.6	48.2
Compound Symmetry *ρ* = 0.7															
-6/-15	0/0	0.8	12.9	0.1	69.5	0.4	79.2	-10.4	79.3	0.6	65.9	0.8	75.7	-9.9	75.5
-6/-15	25/0	-1.1	14.1	0.4	69.9	-16.9	62.4	-18.3	71.0	0.7	65.5	-16.9	57.4	-18.2	66.3
0/0	0/0	0.6	13.4	2.2	72.9	1.8	80.6	1.3	87.4	1.7	68.4	1.6	75.6	1.1	83.7
0/0	25/0	-3.7	13.2	-1.0	69.9	-18.1	61.9	-9.3	79.4	-1.1	65.2	-18.2	57.6	-9.4	74.9
10/25	0/0	2.6	14.1	-0.3	69.1	-0.5	77.5	16.9	95.0	-0.1	64.3	-0.5	72.7	16.2	91.9
10/25	25/0	0.3	13.5	-0.6	70.3	-17.2	62.6	9.5	90.7	-0.7	66.5	-17.3	58.0	7.3	85.1
Toeplitz Covariance															
-6/-15	0/0	-1.5	13.2	0.3	54.9	-0.4	62.3	-10.9	62.2	1.0	52.0	-0.3	78.7	-6.2	83.2
-6/-15	25/0	0.6	13.9	-0.2	53.3	-17.2	46.8	-18.1	54.4	-0.3	49.1	-25.3	53.6	-22.7	66.1
0/0	0/0	0.3	13.3	0.0	54.2	-0.9	62.5	-1.0	71.9	-0.5	51.3	-0.5	78.8	-1.1	87.1
0/0	25/0	1.9	15.5	1.2	56.0	-16.8	46.7	-7.7	64.4	2.0	52.3	-24.6	55.0	-16.6	73.5
10/25	0/0	0.1	14.5	-1.8	52.8	-1.4	61.0	16.5	83.7	-1.4	50.7	-1.2	76.6	9.1	91.2
10/25	25/0	3.5	14.9	1.1	54.5	-16.1	48.0	9.5	77.5	0.8	51.3	-23.8	55.7	-8.0	79.7
Unstructured Covariance															
-6/-15	0/0	0.6	47.3	-1.5	54.7	-0.9	64.3	-11.1	65.4	-0.5	71.8	0.1	85.5	-5.7	85.3
-6/-15	25/0	-0.5	47.5	-0.3	57.1	-17.0	49.8	-18.0	58.2	-0.8	72.2	-15.9	72.1	-16.2	77.0
0/0	0/0	1.9	49.6	-0.6	57.3	-1.1	64.9	-0.7	73.7	-0.3	73.1	-1.1	85.2	-0.7	89.8
0/0	25/0	-1.9	45.4	-0.5	56.5	-18.0	48.2	-8.3	67.1	-0.3	72.5	-16.4	69.0	-11.0	80.2
10/25	0/0	-0.9	47.6	-1.9	54.7	-1.6	64.1	16.2	85.4	-1.4	71.7	-1.2	83.5	8.4	93.6
10/25	25/0	-1.6	46.8	0.7	57.5	-16.8	50.4	9.4	79.9	0.0	72.0	-16.2	69.8	-2.3	85.3

^†^ Period 2/Period 3 effects as proportions of Period 3 standard deviation.

^‡^ T_1_/T_2_ carryover effects as proportions of Period 3 standard deviation.

For the three-treatment crossover trials with a balanced design (Table 5), one-way ANOVA and the PE-CE model with either a CS or an UN covariance structure produced unbiased treatment effect estimates of T_1_ when analyzing the data that were simulated from three different covariance structures including the TP structure. The PE-NCE model and NPE-NCE model with an identical covariance structure generated similar treatment effect estimates, and these estimates were biased when the carryover effects existed. The bias was proportional to the magnitude of the carryover effects. The three GLMs with either a CS or an UN covariance structure were more powerful than was the one-way ANOVA in detecting whether the treatment effect of T_1_ was zero, regardless of the presence of period and carryout effects. The power of these models increased as the within-subject correlation coefficient of the CS covariance structure increased from 0.2 to 0.7.

The GLMs with a CS covariance structure provided slightly larger power than did the corresponding models with an UN covariance structure when analyzing the datasets generated from the CS variance-covariance matrices. However, when the datasets were simulated from the UN covariance structure, misspecification of covariance structure in GLMs reduced the power by more than 20%. For the three-treatment crossover trials with an unbalanced design (Table 6), the same patterns in PE and power were observed for the one-way ANOVA procedure and the three GLMs. A cross-table comparison between the numerical results in Table 5 for the three-treatment crossover trials with a balanced design and the results in Table 6 with an unbalanced design revealed that the power presented in Table 6 was obviously lower than the power in the corresponding position in Table 5.

## Discussion and conclusion

In this research note, we report Monte-Carlo simulation studies on the impact of misspecification of period and carryover effects, as well as covariance structures, in the GLMs with correlated errors for analyzing the data collected from crossover clinical trials. For the two-by-two crossover trials comparing two treatments, the four analysis approaches tested all provide reasonable control of type I error, except for the NPE-NCE model as a misspecified model. The PE-CE model cannot improve power from the naïve two-sample *t*-test that analyzes the data from the first period. Due to model misspecification, the treatment effect estimates given by the PE-NCE and NPE-NCE models are biased when period and carryover effects exist. It should have been plausible to consequently recommend prioritizing the use of the PE-CE models over other approaches. However, in the two-by-two crossover trials, the carryover effects are not identifiable unless further assumptions are made for these effects. In the simulation studies reported in this note, we assume the carryover effect difference is proportional to the treatment effect. Therefore, our simulation results indicate that the advantage of two-by-two crossover design vanishes when carryover effects do. This type of crossover trial is only recommended with prior knowledge that the carryover effects are trivial, in which the PE-NCE models are recommended for data analysis. Using a washout period between two periods is highly encouraged to eliminate carryover effects.

For three-treatment crossover trials with a substantial number of sequences, the GLMs including carryover and period effects (i.e., the PE-CE models) can provide significantly higher empirical power than does the one-way ANOVA approach, by which only the measurements from the first period are tested, and unbiased treatment effect estimates can be attained with this model assuming the UN covariance structures. Otherwise, misspecification in either period effects, carryover effects, or covariance structures can induce inflated type I error, declined power, or biased treatment effect estimates. Therefore, we recommend adopting the PE-CE model with a UN covariance structure for the data analysis in this setting. Additionally, the balanced crossover design should be preferred over the unbalanced crossover design. The numerical results also indicate that, to achieve additional power against the conventional parallel-group study design, the two-period crossover design is recommended only when the carryover effect is negligible, and the three-period crossover design is recommended even when substantial carryover effect exists. However, to make a fair comparison between parallel-group and crossover designs, investigators need to consider costs and duration of the clinical trials.

In this research note, we only considered two-period, two-treatment and three-period, three-treatment crossover trials involving first-order carryover effects. Extrapolation of our results and recommendations beyond this range of design specifications require further investigation and evidence.
